# Angiopoietin-like protein 4 potentiates DATS-induced inhibition of proliferation, migration, and invasion of bladder cancer EJ cells; involvement of G_2_/M-phase cell cycle arrest, signaling pathways, and transcription factors-mediated MMP-9 expression

**DOI:** 10.1080/16546628.2017.1338918

**Published:** 2017-06-20

**Authors:** Seung-Shick Shin, Jun-Hui Song, Byungdoo Hwang, Sung Lyea Park, Won Tae Kim, Sung-Soo Park, Wun-Jae Kim, Sung-Kwon Moon

**Affiliations:** ^a^ Department of Food Science and Nutrition, Jeju National University, Jeju, South Korea; ^b^ Department of Food and Nutrition, Chung-Ang University, Anseong, South Korea; ^c^ Department of Urology, Chungbuk National University, Cheongju, South Korea

**Keywords:** ANGPTL4, bladder cancer, diallyl trisulfide, migration, invasion, microarray

## Abstract

**Background**: Diallyl trisulfide (DATS), a bioactive sulfur compound in garlic, has been highlighted due to its strong anti-carcinogenic activity.

**Objective**: The current study investigated the molecular mechanism of garlic-derived DATS in cancer cells. Additionally, we explored possible molecular markers to monitoring clinical responses to DATS-based chemotherapy.

**Design**: EJ bladder carcinoma cells were treated with different concentration of DATS. Molecular changes including differentially expressed genes in EJ cells were examined using immunoblot, FACS cell cycle analysis, migration and invasion assays, electrophoresis mobility shift assay *(EMSA)*, microarray, and bioinformatics analysis.

**Results**: DATS inhibited EJ cell growth via G_2_/M-phase cell cycle arrest. ATM-CHK2-Cdc25c-p21WAF1-Cdc2 signaling cascade, MAPKs, and AKT were associated with the DATS-mediated growth inhibition of EJ cells. DATS-induced inhibition of migration and invasion was correlated with down-regulated MMP-9 via reduced activation of AP-1, Sp-1, and NF-κB. Through microarray gene expression analysis, ANGPTL4, PLCXD1, and MMP3 were identified as candidates of molecular targets of DATS. Introduction of each gene to EJ cells revealed that ANGPTL4 was associated with the DATS-induced inhibition of cell growth, migration, and invasion.

**Conclusions**: ANGPTL4 regulates DATS-mediated inhibition of proliferation, migration, and invasion of EJ cells, and thus, has potential as a prognostic marker for bladder cancer patients.

## Introduction

Bladder cancer is one of the most common cancers of the human genitourinary system worldwide. According to the American Cancer Society, bladder cancer accounts for approximately 5% of new cancer cases, and in 2016 an estimated 77,000 cases were diagnosed, among which 16,390 patients died due to the disease in the USA [1]. Thus, the development of new therapeutic options is crucial to long-term survival of bladder cancer patients.

Garlic (*Allium sativum*) is renowned as a medicinal food in traditional and alternative medicine. Epidemiological studies support a positive association between intake of *Allium* vegetables and low incidence of cancers [2,3]. When fresh garlic is crushed or chopped, the sulfoxide constituent alliin is enzymatically converted into allicin, which is responsible for the aroma of fresh garlic [4]. Allicin is very unstable and is readily converted into sulfur-containing compounds such as diallyl sulfide (DAS), diallyl disulfide (DADS), diallyl trisulfide (DATS), and other allyl polysulfides [3,5]. Recently, these organic sulfur compounds have attracted great attention as a novel pool of cancer preventive agents [3,5]. For example, a growing body of evidence indicates that DATS inhibits prostate, lung, gastric, and breast cancer progression by inducing apoptosis [6,7].

Cell-cycle progression is regulated by cell-cycle checkpoints at the G1, S, and G_2_/M phases [8]. The G_2_/M checkpoint, which prevents DNA-damaged cells from entering mitosis, is regulated by cyclin-dependent kinase 1 (CDK1), also known as Cdc2, and its activating partner cyclin B1 [9]. Activation of the cyclin B1-CDK1 complex is controlled by either inhibitory phosphorylation of CDK1 by WEE1 and MYT1 kinases or activation of Cdc25c phosphatase by ATM/CHK2 [9]. In addition, chemotherapeutic reagents modulate mitogen-activated protein kinase (MAPK) and AKT cascades, which are key signaling pathways associated with cell death and growth inhibition of bladder cancer cells [10,11]. Furthermore, expression of MMP-9 (gelatinase B, a 92-kDa gelatinase) is closely related with the migration and invasion ability of bladder tumor cells via the activation of transcription factors, including AP-1, Sp-1, and NF-κB [12,13]. Thus, targeting of cell cycle regulation, signaling pathways, and transcription factor-associated MMP-9 modulation might prevent tumor proliferation and metastasis, consequently reducing mortality.

Angiopoietin-like protein 4 (ANGPTL4) is an endogenous inhibitor of lipoprotein lipase that is regulated by fatty acids through PPAR regulatory pathways [14]. Although the major function of ANGPTL4 is to regulate adipogenesis, recent studies have suggested diverse roles in various cancers including colorectal cancer [15], hepatocellular carcinoma (HCC) [16], breast cancer [17], and prostate cancer [18].

Although the inhibitory effects of DATS on cancer cell proliferation have been well demonstrated, the underlying molecular mechanisms remain largely unclear. In this study, we investigated the mechanism of DATS-mediated inhibition of proliferation, migration, and invasion of EJ bladder cancer cells through comprehensive analysis of signaling pathways, cell cycle regulation, and transcription factor-associated MMP-9 regulation. Microarray analysis identified ANGPTL4 as a crucial factor associated with the DATS-mediated anti-tumor effect in EJ cells.

## Methods and materials

### Cells and materials

DATS (SMB00289) was purchased from Sigma-Aldrich (St. Louis, MO, USA). Antibodies were purchased from Santa Cruz Biotechnology (Santa Cruz, CA, USA) or Cell Signaling Technology (Danvers, MA, USA). The nuclear extract kit and electrophoretic mobility shift assay (EMSA) gel shift kit were obtained from Panomics (Fremont, CA, USA). cDNAs of ANGPTL4, PLCXD1, MMP3, and vectors pOTB7 and pCNS were obtained from the Korean Human Gene Bank. The human bladder carcinoma cell line EJ was purchased from the American Type Culture Collection (Manassas, VA, USA). Detailed information on cells and materials is available in the Supporting Information.​​

### DATS treatment and cell counting

EJ cells were seeded in 6-well plates and treated with DATS (0, 50, 100, and 150 µM) for 24 h. The cells were detached from the plates by treatment with 0.25% trypsin containing 0.2% EDTA (Corning, NY, USA). Fifty microliters of detached cells were mixed with 50 μL of 0.4% trypan blue (Sigma-Aldrich) by gentle pipetting, after which 20 μL of the mixture was loaded into each chamber of a hemocytometer, and the cells were counted.

### MTT assay

Cellular proliferation was measured by the MTT (3-(4,5-dimethylthiazol-2-yl)-2,5-diphenyltetrazolium bromide) assay as described previously with some modification [19]. In brief, EJ cells were treated with different concentrations of DATS (0, 50, 100, and 150 μM) for 24 hr. Then, the medium was removed and the cells were incubated with 0.5 mg/mL of MTT solution. After incubation for 2 hr at 37°C in a 5% CO_2_ incubator, the supernatant was removed and 100 uL of DMSO was added. After incubation for 1 hr, cell proliferation was determined by measuring the absorbance at 540 nm on a microplate reader. Cell morphology was analyzed using phase-contrast microscopy.

### Cell-cycle analysis

After treatment with DATS (0, 50, 100, and 150 μM) for 24 hr, the cells were collected and washed twice with 1 × PBS. To determine cell cycle distribution, 5 mL of ice-cold ethanol (70% (*v/v*)) was added dropwise and resuspended the cell pellets and stored at 4°C overnight. The cells were then pelleted and resuspended in 1 ml of 1 × PBS containing RNase A (100 μg/mL) and propidium iodide (100 μg/mL), followed by incubation at 37°C for 30 min. Cell-cycle distribution was analyzed by a flow cytometer (Facstar, Becton Dickinson, Franklin Lakes, NJ, USA) supplied with BD Cell FIT software.

### Wound-healing migration assay

Cells (3 × 10^5^) were plated in 2 mL of medium in each well of a 6-well plate and grown to 90% confluence. The surface area of the cells was scratched with a 2-mm-wide tip. After washing with PBS three times, the plate was incubated with culture media in the presence or absence of DATS for 24 h. The rate of cells migrating into the scratched area was measured and photographed using an inverted microscope at a magnification of 40 ×.

### Boyden chamber invasion assay

Invasion assays were performed using an invasion assay kit (Cell Biolabs, San Diego, USA) according to the manufacturer’s instructions. Cells (2.5 × 10^4^) were suspended in serum-free medium and seeded in the upper chamber. Medium supplemented with 10% FBS was added to the lower chamber as a chemo-attractant. After 24 h of incubation, cells in the lower chamber were stained and photographed.

### Gelatin zymography

Culture medium from EJ cells treated with or without DATS was separated on a polyacrylamide gel containing 1 mg/mL gelatin. The gel was washed with 2.5% Triton X-100 at room temperature for 2 h and subsequently incubated in a buffer containing 10 mM CaCl_2_, 150 mM NaCl, and 50 mM Tris–HCl, pH 7.5 at 37°C overnight. The gel was stained with 0.2% Coomassie blue and photographed. Protease activity of MMPs was detected as white zones.

### Transfection

EJ cells were transfected with each cDNA of ANGPTL4, PLCXD1, MMP3, or empty vector. Briefly, 2 ug of each cDNA was mixed with 1 × OPTI-MEM (Thermo Fisher Scientific), containing 15 uL of Lipofectamine 2000 transfection reagent (Thermo Fisher Scientific). After incubation at room temperature for 5 min, the mixture was carefully added to the cells at 90% confluence in 60 mm culture plates and incubated for 8 hr, followed by changing medium to 1 × DMEM. After incubation for additional 16 h, cells were treated with different concentration of DATS (0, 50, 100, and 150 μM) for the indicated period. Then, the cells were investigated by immunoblotting, FACS, wound-healing migration and Boyden chamber invasion assays, zymography, and EMSA. After incubation for the indicated period, the cells were investigated by immunoblotting, FACS, wound-healing migration and Boyden chamber invasion assays, zymography, and EMSA.

### Nuclear extracts and electrophoretic mobility shift assay (EMSA)

After harvesting, cells were washed and resuspended in a buffer to prepare nuclear extracts for EMSA. For more details, please refer to the Supporting Information.

### RNA extraction

Total RNA was isolated from EJ cells treated with or without DATS using TRIzol reagent (Thermo Fisher Scientific). RNA quality was validated by 1% agarose gel electrophoresis.

### Microarray gene expression profiling and data analysis

Biotin-labeled cRNA for the hybridization was prepared according to the manufacturer’s recommended procedure. For detailed procedures, please refer to the Supporting Information.

### Statistical analysis

Where appropriate, data are represented as the means ± standard errors (SEs). Data were evaluated by factorial ANOVA and Fisher’s least significant difference test where appropriate. Statistical significance was considered at *p* < 0.05.

## Results

### DATS inhibits EJ bladder cancer cell proliferation by inducing cell cycle arrest at G2/M phase

**Figure 1.**
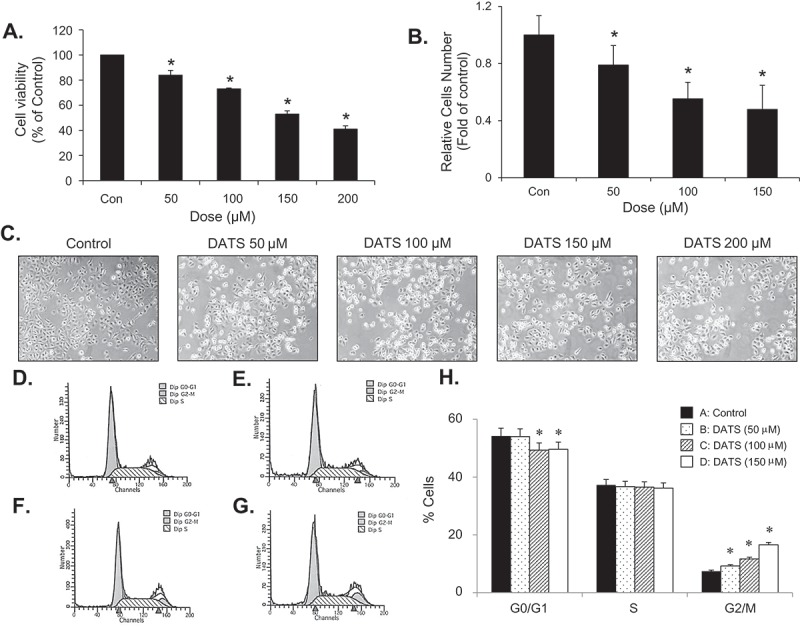
DATS inhibits the proliferation of bladder cancer EJ cells via G_2_/M-phase arrest. (a, b) Cellular viability and proliferation were determined by both MTT assays and cell counting. (c) Images of EJ cells treated with different concentrations of DATS. (d–g) Cell cycle distribution of cells treated with 0 (d), 50 (e), 100 (f), and 150 μM of DATS (g). (h) The percentage of each population in cell cycle phase is presented. Results in bar graphs are expressed as the mean ± SE from three different triplicate experiments. *p < 0.05, vs. control.

To understand the effects of DATS, we treated EJ cells with various concentrations of DATS for 24 h, after which the cytotoxicity and cell growth were measured by both MTT assays (Figure 1(a)) and hemocytometer cell counting (Figure 1(b)). DATS significantly lowered the viability and proliferation rate of EJ cells in a concentration-dependent manner (Figure 1(a,b)). In addition, DATS-treated cells exhibited significant morphological changes and reduced cell density (Figure 1(c)). Based on the results, we selected the concentration of DATS for further experiments to lower than 150 μM, at which the IC_50_ concentration was observed. To examine whether DATS-mediated growth inhibition is associated with cell-cycle arrest, DATS-treated EJ cells were subjected to flow cytometric analysis (Figure 1(d–h)). As concentrations of DATS increased, accumulation at G_2_/M phase increased proportionally (Figure 1(d–h)). These results showed that DATS inhibits proliferation of EJ cells through a mechanism involving G_2_/M phase cell-cycle arrest.

### DATS induces G2/M-phase cell-cycle arrest through the ATM-mediated CHK2/Cdc25c/Cdc2 pathway by upregulating p21WAF1 expression

**Figure 2.**
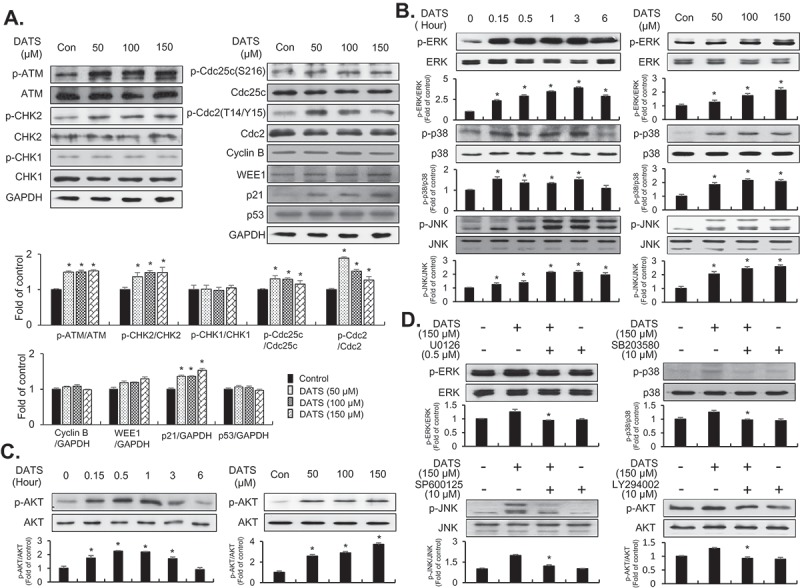
Effects of DATS on the G_2_/M-phase cell cycle regulators and signaling pathways in EJ cells. (a) Protein expression changes induced by DATS were measured by immunoblotting using antibodies specific for the indicated proteins. The results were presented as a fold ratio to the expression of either total form or GAPDH. (b, c) Effects of DATS on MAPK (ERK1/2, JNK1/2, and p38 MAPK) and AKT were determined by immunoblotting. Results are expressed as fold changes compared to the expression of the control. (d) Cells were pre-incubated with U0126 (0.5 μM), SB203580 (10 μM), SP600125 (10 μM), and LY 294,002 (10 μM) for 40 min prior to DATS treatment (150 μM). The ratio of the phosphorylated to the un-phosphorylated form was measured and expressed as fold change compared to DATS treatment.

To examine the effect of DATS on the G_2_/M-checkpoint in EJ cells, the activation of ATM, phosphorylation of CHK1/CHK2 kinases, and inactive phosphorylated forms of Cdc2 kinases were initially analyzed by immunoblotting (Figure 2(a)). DATS treatment (50, 100, and 150 μM for 24 h) induced the activation of ATM and phosphorylation of CHK2 kinases (Figure 2(a), left panel). However, CHK1 phosphorylation was not detected (Figure 2(a), left panel). In addition, p21WAF1 expression and inhibitory phosphorylation of Cdc25c (Ser-216) were increased, whereas the expression levels of p53 and WEE1 remained unchanged (Figure 2(a), right panel). Furthermore, inhibitory phosphorylation of Cdc2 (Thr-14/Tyr-15) was increased in EJ cells after DATS treatment (Figure 2(a), right panel). Interestingly, cyclin B1 was unchanged by DATS treatment (Figure 2(a), right panel). These results supported the flow-cytometric analysis results showing that DATS-induced cell growth retardation was due to G2/M-phase cell cycle arrest in EJ cells.

### DATS treatment leads to phosphorylation of ERK1/2, JNK, and p38 and induces AKT signaling pathways

Several lines of evidence have demonstrated that DATS leads to MAPK phosphorylation [20,21] and AKT signaling pathway activation [22] in cancer cells. To investigate whether DATS affects these pathways in EJ cells, the phosphorylation of ERK1/2, JNK1/2, p38, and AKT kinase was examined by immunoblotting. DATS treatment for up to 6 h led to phosphorylation of ERK1/2, JNK1/2, p38 (Figure 2(b)), and AKT (Figure 2(c)) in a time- and concentration-dependent manner. Phosphorylation of JNK1/2, ERK1/2, p38, and AKT peaked after 1 h or 3 h of DATS treatment (150 μM) (Figure 2(b,c)). To evaluate whether DATS-mediated suppression of proliferation is indeed due to JNK1/2, ERK1/2, p38, and AKT phosphorylation, the cells were treated with SP600125 (an inhibitor of JNK1/2), U0126 (an inhibitor of ERK1/2), SB203580 (an inhibitor of p38), or LY294002 (an inhibitor of AKT) for 1 h followed by treatment with or without DATS for an additional 1 h or 3 h (Figure 2(d)). Pretreatment of EJ cells with the inhibitors reversed the DATS-induced phosphorylation of the respective kinases (Figure 2(d)). Taken together, these results suggested that AKT and MAPK signaling pathways are involved in DATS-induced inhibition of proliferation of bladder cancer EJ cells.

### DATS inhibits migration and invasion of EJ cells via decreased MMP-9 expression by suppressing the binding of transcription factors AP-1, Sp-1, and NF-κB

**Figure 3.**
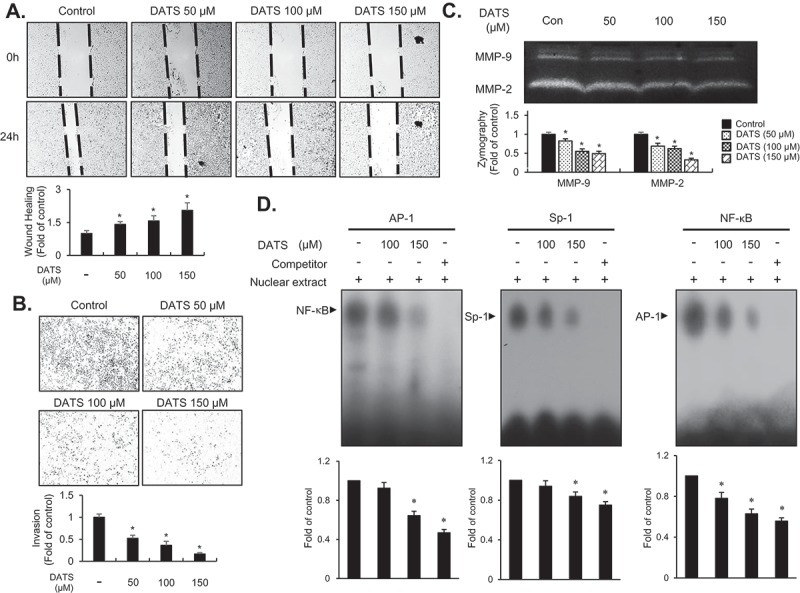
DATS represses the migration and invasion of EJ cells through decreased MMP-9 expression by suppressing of the binding of Sp-1, AP-1, and NF-κB. (a) Wound-healing ability of EJ cells under DATS treatment was assessed by migration assays. The recovery rate was measured as a fold change as compared to the control. (b) Invasion of EJ cells were investigated by invasion assays using Matrigel®-coated chambers. The amount of invading cells was presented as a fold difference relative to the control. (c) Activities of MMP-2 and 9 were assessed by zymography using cell culture medium from DATS-treated cells. Protease activity of each MMP was measured as a fold change compared to the untreated control. (d) The activation of AP-1, Sp-1, and NF-κB was measured by EMSA. Binding activity of transcription factors under each condition was compared to the untreated control and presented as a fold change. Results in bar graphs are presented as the mean ± SE from three different triplicate experiments.

​​The inhibitory effect of DATS on EJ cell metastatic potential was examined using *in vitro* wound healing and invasion assays. The wound-closure and invasive rates were reduced dose-dependently by DATS treatment (Figure 3(a,b)), suggesting that DATS may inhibit the metastatic potential of bladder cancer cells. Matrix metalloproteinases including MMP-9 were previously reported as key regulators in aggressiveness and poor prognosis of bladder cancers [23,24]. Therefore, we examined the activity of MMP-9 in DATS-treated EJ cells by gelatin zymography. As shown in Figure 3(c), the gelatin-degrading activity of MMP-9 was decreased by DATS treatment. Consistent with the results of the wound healing and Boyden chamber assays, inhibition of MMP-9 indicated that DATS might impede the metastatic potential of bladder cancer cells. Previous studies have demonstrated that transcription factors including Sp-1, AP-1, and NF-κB play​​ a key role in the regulation of MMP-9 in cancers [25,26]. Hence, we investigated which transcription factors were involved in the DATS-mediated inhibition of migration and invasion of EJ cells using an EMSA. Binding of all three transcription factors AP-1, Sp-1, and NF-κB​​ was abrogated by DATS treatment (Figure 3(d)). These findings suggested that DATS suppressed the expression of MMP-9 by impeding the binding activities of AP-1, Sp-1, and NF-κB, which resulted in the inhibition of migration and invasion of EJ cells.

### DATS treatment alters gene expression patterns in EJ cells

**Figure 4.**
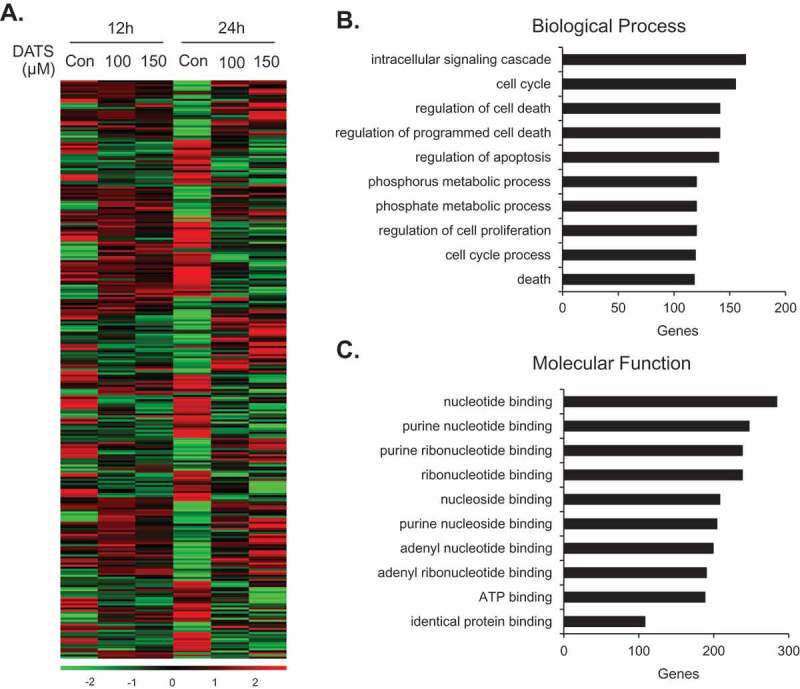
Gene expression patterns of EJ cells induced by DATS. (a) Differential patterns of gene expression were identified by comparative microarray analysis of DATS-treated EJ cells versus untreated cells. The red and green colors indicate high and low gene expression, respectively. (b, c) Differentially expressed genes (2,285 genes) upon DATS treatments were classified by their biological processes (b) and molecular functions (c).

Although the cellular mechanism of DATS has been intensively studied, genomic approaches such as microarray analysis are infrequent. We performed a comparative expression analysis of EJ cells treated with or without DATS (100 and 150 µM, for 12 and 24 h) to identify genes associated with the mechanism of action of DATS using a microarray harboring 47,323 probe sequences derived from RefSeq genes. mRNAs with fold changes ≥1.5 or ≤-1.5 and *p* < 0.05 were regarded differentially expressed. Hierarchical clustering was employed to analyze systematic variations in mRNA expression in DATS-treated groups as compared to untreated groups. DATS treatment led to differential expression of 2,285 genes (*p*  <  0.05), of which 1,121 were upregulated and 1,164 were downregulated (Figure 4(a)). The genes were functionally categorized using the DAVID database. To identify functional processes of biological importance, we analyzed the enrichment of Gene Ontology (GO) terms in the ‘Biological Process’ (‘BP’) and ‘Molecular Function’ (‘MF’) categories of differentially expressed genes. Figure 4(b) shows the most significantly enriched terms in BP. In this category, the 10 most highly upregulated genes in the DATS-treated (100 and 150 µM; 24 h) versus untreated groups were *IL1B, HMOX1, SPHK1*, *GADD45A, SERPINB2*, *ANGPTL4, SPSB1*, *ITGA2, HBEGF*, and *PLCXD1* (Table 1). ​​The 10 most downregulated genes were *THBS1*, *CCL2, DEPDC6*, *NR4A2, TNFRSF6B*, *EFNA1, E2F2*, *TRIB2, PDGFB*, and *TNFRSF11B* (Table 2). ​​Figure 4(c) shows the most significantly enriched terms in MF. In this category, the 10 most highly upregulated genes were *HSPA6*, *SPHK1, SGK DAPK3, TXNRD1*, *MGC87042, SCHIP1*, *RGMB, FHL2*, and *ITGA2*, and the 10 most downregulated genes were *TNFRSF6B*, *SIK1, TRIB2*, *IKBKE, ADORA1*, *DHRS3, THBS1*, *PDGFB, CLDN16*, and *CLDN11* (Tables 3 and 4). ​​Finally, the 15 most upregulated were identified using a fold-change filter (Table 5). ​​Among these, *ANGPTL4, PLCXD1*, and *MMP3*, for which cDNAs were available in the Korean Human Gene Bank, were selected for further investigation. Immunoblot analysis confirmed that ANGPTL4, PLCXD1, and MMP3 were upregulated by DATS treatment, corroborating the microarray results (Figure S1A).​​

**Table 1.** The 10 most upregulated genes in the BP category following DATS treatment.

Symbol	Description	Go Term
*IL1B*	Interleukin 1, beta	Intracellular signaling cascade
		Regulation of programmed cell death
		Regulation of cell death
		Regulation of apoptosis
		Regulation of cell proliferation
		Phosphate metabolic process
		Phosphorus metabolic process
		Death
*HMOX1*	Heme oxygenase (decycling) 1	Intracellular signaling cascade
		Regulation of programmed cell death
		Regulation of cell death
		Regulation of apoptosis
		Regulation of cell proliferation
		Death
*SPHK1*	Sphingosine kinase 1	Intracellular signaling cascade
		Regulation of programmed cell death
		Regulation of cell death
		Regulation of apoptosis
		Regulation of cell proliferation
*GADD45A*	Growth arrest and DNA-damage-inducible, alpha	Cell cycle
		Cell cycle process
		Death
*SERPINB2*	Serpin peptidase inhibitor, clade B (ovalbumin), member 2	Regulation of cell death
		Regulation of apoptosis
		Regulation of programmed cell death
*ANGPTL4*	Angiopoietin-like 4	Regulation of cell death
		Regulation of apoptosis
		Regulation of programmed cell death
*SPSB1*	SplA/ryanodine receptor domain and SOCS box containing 1	Intracellular signaling cascade
*ITGA2*	Integrin, alpha 2 (CD49B, alpha 2 subunit of VLA-2 receptor)	Regulation of cell proliferation
*HBEGF*	Heparin-binding EGF-like growth factor	Regulation of cell proliferation
*PLCXD1*	Phosphatidylinositol-specific phospholipase C, X domain containing 1	Intracellular signaling cascade

**Table 2.** The 10 most downregulated genes in the BP category following DATS treatment

Symbol	Description	Go Term
*THBS1*	Thrombospondin 1	Intracellular signaling cascade
		Cell cycle
		Regulation of programmed cell death
		Regulation of cell death
		Regulation of apoptosis
		Regulation of cell proliferation
		Phosphate metabolic process
		Phosphorus metabolic process
		Cell cycle process
		Death
*CCL2*	Chemokine (C-C motif) ligand 2	Intracellular signaling cascade
		Regulation of programmed cell death
		Regulation of cell death
		Regulation of apoptosis
		Regulation of cell proliferation
		Phosphate metabolic process
		Phosphorus metabolic process
*DEPDC6*	DEP domain containing 6	Regulation of cell death
		Regulation of apoptosis
		Intracellular signaling cascade
		Regulation of programmed cell death
*NR4A2*	Nuclear receptor subfamily 4, group A, member 2	Regulation of programmed cell death
		Regulation of cell death
		Regulation of apoptosis
		Death
*TNFRSF6B*	Tumor necrosis factor receptor superfamily, member 6b,	Regulation of programmed cell death
		Regulation of cell death
		Regulation of apoptosis
		Death
*EFNA1*	Ephrin-A1	Intracellular signaling cascade
		Phosphate metabolic process
		Phosphorus metabolic process
*E2F2*	E2F transcription factor 2	Cell cycle
		Death
*TRIB2*	Tribbles homolog 2 (Drosophila)	Phosphate metabolic process
		Phosphorus metabolic process
*PDGFB*	Platelet-derived growth factor beta polypeptide	Regulation of cell proliferation
*TNFRSF11B*	Tumor necrosis factor receptor superfamily, member 11b	Death

**Table 3.** The 10 most upregulated genes in the MF category following DATS treatment

Symbol	Description	Go Term
*HSPA6*	Heat shock 70-kDa protein 6 (HSP70B')	Nucleotide binding
		Purine nucleotide binding
		Ribonucleotide binding
		Purine ribonucleotide binding
		Nucleoside binding
		Purine nucleoside binding
		Adenyl nucleotide binding
		Adenyl ribonucleotide binding
		ATP binding
*SPHK1*	Sphingosine kinase 1	Nucleotide binding
		Purine nucleotide binding
		Ribonucleotide binding
		Purine ribonucleotide binding
		Nucleoside binding
		Purine nucleoside binding
		Adenyl nucleotide binding
		Adenyl ribonucleotide binding
		ATP binding
*SGK*	Serum/glucocorticoid regulated kinase 1	Nucleotide binding
		Purine nucleotide binding
		Ribonucleotide binding
		Purine ribonucleotide binding
		Nucleoside binding
		Purine nucleoside binding
		Adenyl nucleotide binding
		Adenyl ribonucleotide binding
		ATP binding
*DAPK3*	Death-associated protein kinase 3	Nucleotide binding
		Purine nucleotide binding
		Ribonucleotide binding
		Purine ribonucleotide binding
		Nucleoside binding
		Purine nucleoside binding
		Adenyl nucleotide binding
		Adenyl ribonucleotide binding
		ATP binding
*TXNRD1*	Thioredoxin reductase 1	Adenyl nucleotide binding
		Nucleotide binding
		Purine nucleotide binding
		Nucleoside binding
		Purine nucleoside binding
*MGC87042*	Six transmembrane epithelial antigen of the prostate 1	Adenyl nucleotide binding
		Nucleotide binding
		Purine nucleotide binding
		Nucleoside binding
		Purine nucleoside binding
*SCHIP1*	Schwannomin interacting protein 1	Identical protein binding
*RGMB*	RGM domain family, member B	Identical protein binding
*FHL2*	Four and a half LIM domains 2	Identical protein binding
*ITGA2*	Integrin, alpha 2	Identical protein binding

**Table 4.** The 10 most downregulated genes in the MF category following DATS treatment

Symbol	Description	Go Term
*TNFRSF6B*	Tumor necrosis factor receptor superfamily, member 6b	Nucleotide binding
		Purine nucleotide binding
		Ribonucleotide binding
		Purine ribonucleotide binding
		Nucleoside binding
		Purine nucleoside binding
		Adenyl nucleotide binding
		Adenyl ribonucleotide binding
		ATP binding
*SIK1*	Salt-inducible kinase 1	Nucleotide binding
		Purine nucleotide binding
		Ribonucleotide binding
		Purine ribonucleotide binding
		Nucleoside binding
		Purine nucleoside binding
		Adenyl nucleotide binding
		Adenyl ribonucleotide binding
		ATP binding
*TRIB2*	Tribbles homolog 2 (Drosophila)	Nucleotide binding
		Purine nucleotide binding
		Ribonucleotide binding
		Purine ribonucleotide binding
		Nucleoside binding
		Purine nucleoside binding
		Adenyl nucleotide binding
		Adenyl ribonucleotide binding
		ATP binding
*IKBKE*	Inhibitor of kappa light polypeptide gene enhancer in B-cells, kinase epsilon	Nucleotide binding
		Purine nucleotide binding
		Ribonucleotide binding
		Purine ribonucleotide binding
		Nucleoside binding
		Purine nucleoside binding
		Adenyl nucleotide binding
		Adenyl ribonucleotide binding
		ATP binding
*ADORA1*	Adenosine A1 receptor	Nucleoside binding
		Purine nucleoside binding
*DHRS3*	Dehydrogenase/reductase member 3	Nucleotide binding
*THBS1*	Thrombospondin 1	Identical protein binding
*PDGFB*	Platelet-derived growth factor beta polypeptide	Identical protein binding
*CLDN16*	Claudin 16	Identical protein binding
*CLDN11*	Claudin 11	Identical protein binding

**Table 5.** Upregulated genes following DATS treatment (150 μM)

Gene name	NCBI Genebank accession no.	Fold change	
		12 h	24 h
*ANGPTL4*	NM_139314.1	4.957095	3.672516
*ESM1*	NM_007036.3	5.676903	4.787466
*GLIPR1*	NM_006851.1	4.27405	7.92755
*HBGF*	NM_001945.1	6.339261	5.412916
*IL1B*	NM_000576.2	3.709475	4.221266
*KRT34*	NM_021013.3	4.629404	2.718188
*LOC100132564*	XM_001713808.1	3.272407	8.328955
*MMP3*	NM_002422.3	6.141729	8.827352
*PLCXD1*	NM_018390.1	6.036078	2.08199
*PPIF*	NM_005729.3	2.285843	4.104856
*PSG4*	NM_002780.3	2.659419	3.961507
*RN7SK*	NR_001445.1	3.159532	4.830147
*SPOCD1*	NM_144569.3	4.383148	3.875434
*TXNRD1*	NM_003330.2	4.987633	3.606279
*TXNRD1*	NM_182743.1	4.607666	2.382806

### Overexpression of ANGPTL4 enhances DATS-induced inhibition of EJ cell proliferation

**Figure 5.**
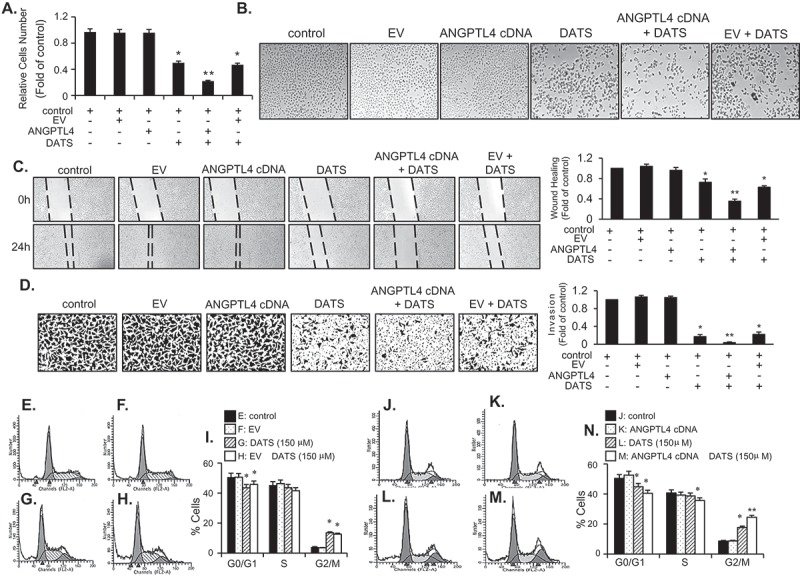
ANGPTL4 enhances the DATS-induced inhibition of proliferation, migration, and invasion and induction of G_2_/M-phase cell cycle arrest in EJ cells. (a) The relative cell number was determined as a fold change compared to the control. (b) Images of cells under different transfection conditions as indicated were photographed under a phase contrast microscope. (c, d) After transfection, wound-healing capacity and invasiveness of EJ cells under the indicated conditions were presented as a fold change compared to the control. (E-N) After transfection with either ANGPTL4 or an empty vector (EV), changes in the cell-cycle phase distribution in response to DATS were analyzed by flow cytometry. The results in bar graphs are presented as the mean ± SE from three different triplicate experiments. *p < 0.05, vs. control and **p < 0.05, vs. DATS treatment.

Because ANGPTL4, PLCXD1, and MMP3 were upregulated by DATS treatment, we aimed to evaluate whether introduction of these genes would affect the proliferation of EJ cells. To this end, EJ cells were transfected with ANGPTL4, PLCXD1, or MMP3 cDNA or an empty vector (EV) as a control, followed by treatment with DATS. The transfection efficiency of the three genes was confirmed by immunoblotting (Figure S1B). ​​As shown in Figure 5(a), no effect on proliferation was observed in ANGPTL4-, PLCXD1-, and MMP3-transfected cells without DATS treatment. Similarly, the EV did not affect proliferation as compared to the no-treatment control (Figure 5(a)). However, interestingly, overexpression of ANGPTL4 cDNA potentiated the inhibitory effect of DATS on EJ cell proliferation, as compared with the DATS-treated controls and DATS-treated EV transfectants (Figure 5(a)). In contrast, the DATS inhibitory effect was not enhanced in either PLCXD1- or MMP3-transfected cells (Figures S1C and D). ​​Morphological analysis of the transfectants supported the notion that ANGPTL4 potentiated DATS-induced inhibition of proliferation of EJ cells (Figure 5(b)), while PLCXD1 and MMP3 did not show such an effect (Figures S1C–F). ​​Based on these results, we concluded that overexpression of ANGPTL4 further fortifies the suppressive effect of DATS against EJ cell proliferation, although ANGPTL4 is not directly involved in proliferation in EJ cells.

### Overexpression of ANGPTL4 enhances DATS-induced inhibition of EJ cell migration and invasion

To investigate whether ANGPTL4 participates in the DATS-mediated inhibitory action on migration and invasion of EJ cells, we transfected cells with ANGPTL4 cDNA or EV followed by DATS treatment. As demonstrated in Figure 5(c,d), overexpression of ANGPTL4 alone did not affect the migration and invasion of EJ cells. However, the DATS-mediated inhibitory effect was significantly potentiated by transfection of ANGPTL4 (Figure 5(c,d)). In contrast, cells transfected with PLCXD1, MMP3, or EV showed no potentiation effect as compared to DATS treatment alone (Figures S2A–D).​​

### Overexpression of ANGPTL4 augments expression of G2/M phase cell cycle-associated proteins and phosphorylation of DATS-induced signaling in EJ cells

**Figure 6.**
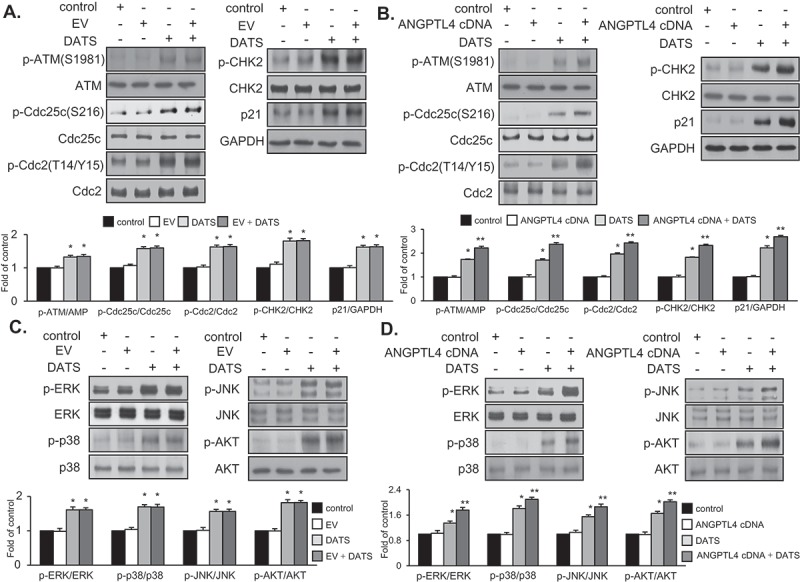
Effect of ANGPTL4 on the regulatory proteins participating in DATS-induced signaling cascades. EJ cells were transfected with either an EV (a, c) or ANGPTL4 cDNA (b, d) followed by incubation in either DATS-containing (150 μM) or normal medium for 24 h. Changes in the protein expression levels as compared to the control were measured by immunoblotting using specific antibodies indicated. Expression was normalized to total forms or GAPDH. Relative fold changes as compared to the control are presented in the graph. *p < 0.05, vs. control and **p < 0.05, vs. DATS treatment.

Based on the results (Figure 5(a–d)), we performed flow cytometric analysis to verify whether the transfection of ANGPTL4 cDNA elicits any change in the distribution of the cell cycle phases. Following transfection, DATS treatment led to an accumulation of EJ cells in the G_2_/M phase as compared to DATS alone (Figure 5(j–n)). On the contrary, EV transfectants showed no changes (Figure 5(e–i)). Next, we investigated whether DATS-mediated G_2_/M phase-associated proteins are altered upon the introduction of ANGPTL4 cDNA into EJ cells. DATS-induced phosphorylation of ATM/CHK2/Cdc25c/Cdc2 signaling was elevated by overexpression of ANGPTL4 (Figure 6(b)) when compared with DATS alone (Figure 6(a)). Accordingly, increased p21WAF1 expression by DATS was further enhanced in ANGPTL4-transfected EJ cells (Figure 6(b)). Next, the effect of ANGPTL4 on MAPK and AKT signaling pathways was investigated by immunoblotting. Interestingly, phosphorylation of ERK1/2, p38, JNK, and AKT in response to DATS was significantly upregulated by transfection of ANGPTL4 as compared to DATS alone (Figure 6(d)), but not EV transfectants (Figure 6(c)). These results suggested that ANGPTL4 must be involved in the inhibition of cell proliferation via the regulation of the G_2_/M phase and the modulation of signaling pathways in DATS-treated EJ cells.

### ANGPTL4 overexpression intensifies the decreased expression of MMP-9 via diminished binding activity of AP-1, Sp-1, and NF-κB in EJ cells

**Figure 7.**
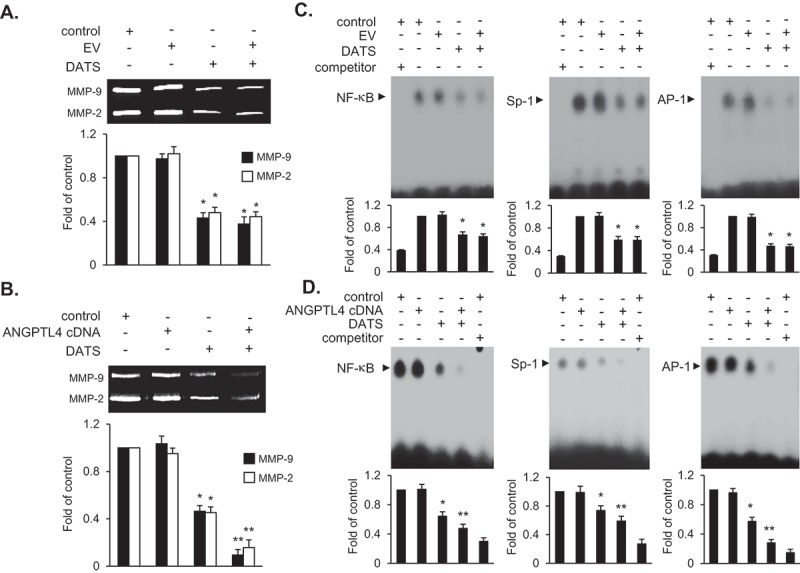
ANGPTL4 potentiates the inhibitory activity of MMP-9 expression via the reduction of binding activities of AP-1, Sp-1, and NF-κB in DATS-treated EJ cells. Cells were transfected with either an EV or ANGPTL4 cDNA. Then, the cells were treated with DATS (150 μM) for 24 h. (a, b) Zymography for MMP-9 expression was carried out using cultured medium. Proteolytic activity of MMP-9 was measured as the fold change compared to the control. (c, d) Binding activities of AP-1, Sp-1, and NF-κB were measured by EMSA using radiolabeled oligonucleotide probes. As competitors, unlabeled AP-1, Sp-1, and NF-κB oligonucleotides were used. Relative fold changes were presented compared to the control. In each bar graph, results are presented as the mean ± SE from three different triplicate experiments. *p < 0.05, vs. control and **p < 0.05, vs. DATS treatment.

​​Finally, we examined the effect of ANGPTL4 on DATS-mediated inhibition of cell migration and invasion by monitoring MMP-9 activity using gelatin zymography. MMP-9 expression in EJ cells transfected with ANGPTL4 cDNA followed by DATS treatment was further decreased at 24 h as compared to DATS treatment alone or DATS treatment of cells transfected with EV (Figure 7(a,b)). EMSA showed that downregulated activities of transcription factors by DATS were also potentiated following the transfection of ANGPTL4 in EJ cells (Figure 7(d)) as compared to EV transfectants (Figure 7(c)). These results demonstrated that ANGPTL4 might be an important regulatory factor in the control of decreased MMP-9 expression by suppressing the binding activities of AP-1, Sp-1, and NF-κB​​ in DATS-treated bladder cancer cells.

## Discussion

In the present study, we investigated how DATS regulates the cellular physiology of EJ bladder cancer cells. In addition, we generated a comprehensive dataset of molecular targets of DATS using microarray technology. Finally, based on the microarray analysis, we identified ANGPTL4 as a key regulatory factor of DATS-mediated inhibition of proliferation, migration, and invasion of EJ cells.

Previous studies have shown that cancer cells treated with DATS are arrested at G_2_ as well as prometaphase stages [3,6,7]. Inter alia, the DATS-mediated prometaphase arrest is caused by checkpoint kinase 1-mediated inactivation of anaphase-promoting complex/cyclosome through accumulation of its substrates [27]. A recent study indicated that cells arrested in prometaphase upon treatment with DATS are ultimately driven to apoptosis [28]. However, the molecular mechanism underlying DATS-mediated G_2_/M arrest in cancer cells was largely unknown. Therefore, we focused on filling in this knowledge gap using the EJ bladder cancer cell line. First, we observed that DATS treatment significantly inhibited the proliferation of the EJ cells through G_2_/M cell-cycle arrest. G_2_/M transition is regulated by Cdc2, whose activity is dependent upon its association with regulator cyclin B1 [29]. However, unexpectedly, the expression level of cyclin B1 in EJ cells was not altered by DATS treatment. A previous study showed that DADS, but not DATS, treatment induced downregulation of cyclin B1 in human gastric cancers and esophageal squamous carcinoma cells [30,31]. On the other hand, similar to our results, PC-3 prostate cancer cells treated with DATS showed no change in cyclin B1 expression [27]. In addition, treatment with DATS induced the activation of ATM and phosphorylation of CHK2 kinases, which brings about the inhibitory phosphorylation of Cdc25c on Ser-216 and upregulation of p21WAF1 expression. Inhibitory phosphorylation of Cdc2 on Thr-14 and Tyr-15 was indeed increased. Collectively, our data demonstrated that DATS induced cell-cycle arrest at G_2_/M, which was dictated by the inhibition of cell growth-mediated activation of the ATM-CHK2-Cdc25c-p21WAF1 pathway, resulting in Cdc2 activation.

The importance of MAPK and AKT signaling pathways in regulating cell proliferation under stress conditions has been widely investigated [10,11]. Previous studies have shown that the MAPK pathway is involved in cell growth inhibition and/or cell cycle regulation [10,11]. Chen and colleagues have reported that all three MAPK are activated in HepG2 hepatoma cells by DATS treatment [20]. In addition, recent research has shown that the activation of AKT is involved in fucoidan-induced growth inhibition in human bladder cancer cells [10]. In agreement with these findings, we observed that DATS treatment resulted in the upregulation of early signal transductions of JNK, ERK1/2, MAPK p38, and AKT. This result suggests that DATS suppresses cell proliferation via the phosphorylation of MAPK and AKT in bladder cancer EJ cells.

It is well known that metastasis is a major factor in disease progression, which accounts for the majority of cancer deaths [12,13]. Previous studies have proposed that the targeting of migration and invasion of tumor cells would be a good strategy to treat muscle-invasive bladder cancer [12,13,26]. Accordingly, we attempted to verify whether DATS treatment inhibited migration and invasion of bladder cancer EJ cells. Previous reports have demonstrated that MMP-9 is associated with the migration and invasion processes of bladder tumors [12,25,32]. Our results showed that the inhibitory activity of DATS in migration and invasion of EJ cells was associated with the inhibition of MMP-9. Previously, it has been shown that diallyl sulfide abolished the expression levels of MMP-9 in Colo205 human colon cancer cells [33]. Additionally, it has been reported that MMP-9 expression is regulated by transcription factors AP-1, Sp-1, and NF-κB [25,26]. Consistent herewith, our study showed that treatment with DATS markedly downregulated the binding activities of AP-1, Sp-1, and NF-κB, which led to the suppression of migration and invasion of EJ cells.

Although the inhibitory effect of DATS on the proliferation and metastasis of bladder cancer cells has been demonstrated, the involved molecular players remained largely unidentified. Therefore, we utilized microarray analysis to identify differentially expressed genes upon DATS treatment. After gene expression profiling of DATS-treated and untreated groups, we selected the 20 most highly up- and downregulated genes in the categories of BP and MF (Tables 1–4). These 20 biologically functional genes might be novel factors associated with the inhibitory mechanism of DATS in proliferation, migration, and invasion of EJ bladder cancer cells. In addition, based on a fold-change filter, we identified 15 genes that were differentially upregulated by DATS treatment (Table 5). From three of these genes (*ANGPTL4*, *PLCXD1*, and *MMP3*) selected for further analysis, we found that ANGPTL4 participates in the inhibition of proliferation, migration, and invasion induced by DATS in EJ cells. Despite the fact that the effects of this protein in tumorigenesis and tumor suppression have been controversial, our data indicated that ANGPTL4 alone exhibits no effect on growth, migration, and invasion of EJ cells. Moreover, we verified that ANGPTL4 is involved in the ATM/CHK2/Cdc25c/p21WAF1/Cdc2 cascade, which led to the G2/M-phase cell-cycle arrest. We also confirmed that ANGPTL4 is a critical cofactor implicated in the MAPK (ERK1/2, JNK, p38) and AKT signaling pathways in the DATS-mediated growth inhibition of EJ cells. In addition, our data demonstrated that ANGPTL4 further enhanced the inhibitory effect of DATS on MMP-9 expression by further suppressing NF-κB, AP-1, and Sp-1 activities. Thus, this study identified ANGPTL4 as a novel potential regulatory factor in the suppression of proliferation, migration, and invasion of DATS-treated bladder carcinoma EJ cells.

## Conclusion

Taken together, ANGPTL4 might be one of the upstream regulators in DATS-mediated signaling cascades, and thus a prognostic marker for DATS-treated bladder cancer patients associated with muscle invasiveness. Further studies should examine the roles of other genes in the inhibition of proliferation, migration, and invasion of bladder cancer cells following DATS treatment.

## Supplementary Material

ZFNR_A_1338918_Supplemental_data.zip
