# Supplementary material for: Angiopoietin-like protein 4 potentiates DATS-induced inhibition of proliferation, migration, and invasion of bladder cancer EJ cells; involvement of G2/M-phase cell cycle arrest, signaling pathways, and transcription factors-mediated MMP-9 expression
Source: Food Nutr Res. 2017 Jun 20;61(1):1338918. doi: 10.1080/16546628.2017.1338918 (PMC5492081; doi:10.1080/16546628.2017.1338918)
Supplement: ZFNR_A_1338918_Supplemental_data.zip [file zfnr_a_1338918_sm0516.zip › ZFNR_A_1338918_Supplemental data/DATS_EJ_Supplementary_information.docx]

**SUPPLEMENTARY INFORMATION**

**Supplementary methods**

*Materials*

Polyclonal antibodies against p21WAF1 (sc-756), p53 (sc-126), p27 (sc-528), p-Cdc2 p34 (sc-12340-R), Cdc2 p34 (sc-54), Cdc25c (sc-327), p-Cdc25c (sc12354), cyclin A (sc-751), cyclin B1 (sc-245), WEE1 (sc-325), CHK2 (sc-9064), p-ATM (sc-47739), ATM (sc-23921), ANGPTL4, PLCXD1, MMP3, and GAPDH (sc-20357) were obtained from Santa Cruz Biotechnology (Santa Cruz, CA, USA). Polyclonal antibodies against p-CHK2 (2661), CHK1 (2360) p-CHK1 (2341) ERK (9102), p-ERK (9101), p38 MAPK (9212), p-p38 MAPK (9211), JNK (9258), p-JNK (9251), AKT (9272), and p-AKT (9271) were purchased from Cell Signaling Technology (Danvers, MA, USA). Goat anti-rabbit IgG-horseradish peroxidase (HRP) (sc-2004), goat anti-mouse IgG-HRP (sc-2005), and donkey anti-goat IgG-HRP (sc-2020) were purchased from Santa Cruz Biotechnology. Western Lightning Plus-ECL was obtained from PerkinElmer (MA, USA). Nuclear Extract and EMSA Gel Shift kits were obtained from Panomics (Fremont, CA, USA). cDNAs of ANGPTL4, PLXCD1, MMP3 and vectors, pOTB7 and pCNS, were obtained from Korea human gene bank.

*Cell cultures*

EJ cells were maintained in Dulbecco’s modified Eagle’s medium (DMEM) (4.5 g glucose/L) supplemented with 10% fetal calf serum, l-glutamine, and antibiotics (Biological Industries, Beit Haemek, Israel) at 37°C in a 5% CO_2_ humidified incubator.

*Immunoblots*

Protein lysates were prepared and quantified as described previously (1). Briefly, lysates were separated by 10% SDS-PAGE under denaturing conditions and transferred to nitrocellulose membranes (Hybond; GE Healthcare Bio-Sciences, Marlborough, MA, USA) followed by blocking with 5% (w/v) non-fat dry milk in TBS (10 mM Tris-HCl (pH 8.0), 150 mM NaCl) and incubation with primary antibodies at 4°C overnight. Then, the blots were incubated with peroxidase-conjugated secondary antibodies for 90 min. Chemiluminescence reagent (GE Healthcare Bio-Sciences) was utilized for the detection of protein complexes. The experiments were repeated at least 3 times.

*Nuclear extracts and electrophoretic mobility shift assay (EMSA)*

After harvest by centrifugation, cells were washed and resuspended in a buffer containing 10 mM HEPES (pH 7.9), 10 mM KCl, 1 mM DTT, 0.5 mM PMSF, 0.1 mM EDTA, and 0.1 mM EGTA. After incubation on ice for 15 min, the cells were vigorously mixed with 0.5% NP-40. The nuclear pellet was collected by centrifugation followed by extraction in a buffer containing 20 mM HEPES (pH 7.9), 400 mM NaCl, 1 mM DTT, 1 mM PMSF, 1 mM EDTA, and 1 mM EGTA at 4°C for 15 min. The nuclear extract (10–20 μg) was preincubated at 4°C for 30 min with a 100-fold excess of an unlabeled oligonucleotide spanning the -79 position of the MMP-9 cis element of interest. The sequences were as follows: AP-1, CTGACCCCTGAGTCAGCACTT; NF-κB, CAGTGGAATTCCCCAGCC; and Sp-1, GCCCATTCCTTCCGCCCCCAGATGAAGCAG. Then, the reaction mixture was incubated at 4°C for 20 min in a buffer (25 mM HEPES buffer (pH 7.9), 0.5 mM EDTA, 0.5 mM DTT, 50 mM NaCl, and 2.5% glycerol) with 2 μg of poly dI/dC and 5 fmol (2 × 10^4^ cpm) of a Klenow end-labeled (32^P^ ATP) 30-mer oligonucleotide, which spanned the DNA-binding site of the MMP-9 promoter. The reaction mixture was separated by electrophoresis at 4°C using a 6% polyacrylamide gel. The gel was exposed to X-ray film overnight.

*Microarray gene expression profiling*

Biotin-labeled cRNA for the hybridization was prepared according to the manufacturer’s recommended procedure. First, cDNA was synthesized from 500 ng of total RNA with several amplification and labeling procedures to prepare biotin-labeled cRNA utilizing an IlluminaH TotalPrep RNA Amplification kit (Ambion, Austin, TX). The cRNA was quantified with a Quant-iT^TM^ RiboGreenH RNA assay kit (Invitrogen-Molecular Probes, ON, Canada) using a Victor3 spectrophotometer (PerkinElmer, CT). cRNA quality was measured by 1% agarose gel electrophoresis and then, biotin-labeled cRNAs (1,500 ng per array) were hybridized to an Illumina Human-6 BeadChip (48K), version 2 (Illumina, San Diego, CA) according to the manufacturer’s protocols. Amplified array signals were detected using Amersham fluorolink streptavidin-Cy3 (GE Healthcare Bio-Sciences, Little Chalfont, UK) following the instructions in the BeadChip manual. The signals from the arrays were scanned on an Illumina Bead Array Reader confocal scanner (BeadStation 500GXDW; Illumina, San Diego, CA).

*Statistical analysis of microarray data*

To compare gene expression patterns between DATS-treated and untreated groups, we utilized hierarchical clustering analysis as described previously (2). Genes showing a *p*-value <0.001 were considered significantly differentially expressed. To survey biological functions within differentially expressed genes, we utilized the Ingenuity^TM^ Pathways Analysis software, which classifies genes by function. The significance of each function was evaluated by the Ingenuity Pathway Analysis Tool (version 8.8).

**Supplementary figure legends**

**Figure S1.** Effect of PLCXD1 and MMP3 overexpression on DATS-induced inhibition of proliferation of EJ cells. (A) EJ cells were treated with DATS (150 μM) for indicated times. Changes in protein expression in response to DATS treatment were confirmed by immunoblot assays using specific antibodies for ANGPLT4, PLCXD1, and MMP3. GAPDH was used as a loading control. The bar graph shows relative fold changes in protein expression levels as compared to expression of each protein at 0 h (control). **p* < 0.05, vs. control. (B) Transfection efficiency was determined by immunoblot assays after transfection of EJ cells with either an EV (pOTB7 or pCNS) or the indicated plasmid (ANGPLT4, PLCXD1, or MMP3). Expression levels were normalized to GAPDH. Fold changes were presented as bar charts. **p* < 0.05, vs. vector control. (C, D) Effect of PLCXD1 or MMP3 in the absence or presence of DATS in EJ cells. Cells were transfected with either an EV or the indicated cDNA followed by incubation in medium with or without DATS (150 μM) for 24 h. Relative cell growth was compared to the control. **p* < 0.05, vs. control. (E, F) Images under different transfection conditions as indicated were obtained under a phase-contrast microscope.

**Figure S2.** Effect of PLCXD1 and MMP3 overexpression on DATS-induced inhibition of migration and invasion of EJ cells. EJ cells were transfected with an EV, or a PLCXD1 or MMP3 expression vector and stimulated with DATS (150 μM) for 24 h. (A, B) Wound healing capacity of EJ cells is presented as a fold change compared to the control. **p* < 0.05, vs. each control. (C, D) Invasiveness of EJ transfectants with PLCXD1, MMP3, or an EV was determined by Boyden chamber assays. Invasiveness of each transfectant was represented as a fold change compared to the control. **p* < 0.05, vs. control.

**Supplementary references**

1. Park SL, Cho TM, Won SY, Song JH, Noh DH, Kim WJ, et al. MicroRNA-20b inhibits the proliferation, migration and invasion of bladder cancer EJ cells via the targeting of cell cycle regulation and Sp-1-mediated MMP-2 expression. Oncol Rep. 2015 Sep;34(3):1605-12.

2. Lee SJ, Lee EJ, Kim SK, Jeong P, Cho YH, Yun SJ, et al. Identification of pro-inflammatory cytokines associated with muscle invasive bladder cancer; the roles of IL-5, IL-20, and IL-28A. PLoS One. 2012;7(9):e40267.
