# Supplementary figures and images for: Angiopoietin-like protein 4 potentiates DATS-induced inhibition of proliferation, migration, and invasion of bladder cancer EJ cells; involvement of G2/M-phase cell cycle arrest, signaling pathways, and transcription factors-mediated MMP-9 expression
Source: Food Nutr Res. 2017 Jun 20;61(1):1338918. doi: 10.1080/16546628.2017.1338918 (PMC5492081; doi:10.1080/16546628.2017.1338918)

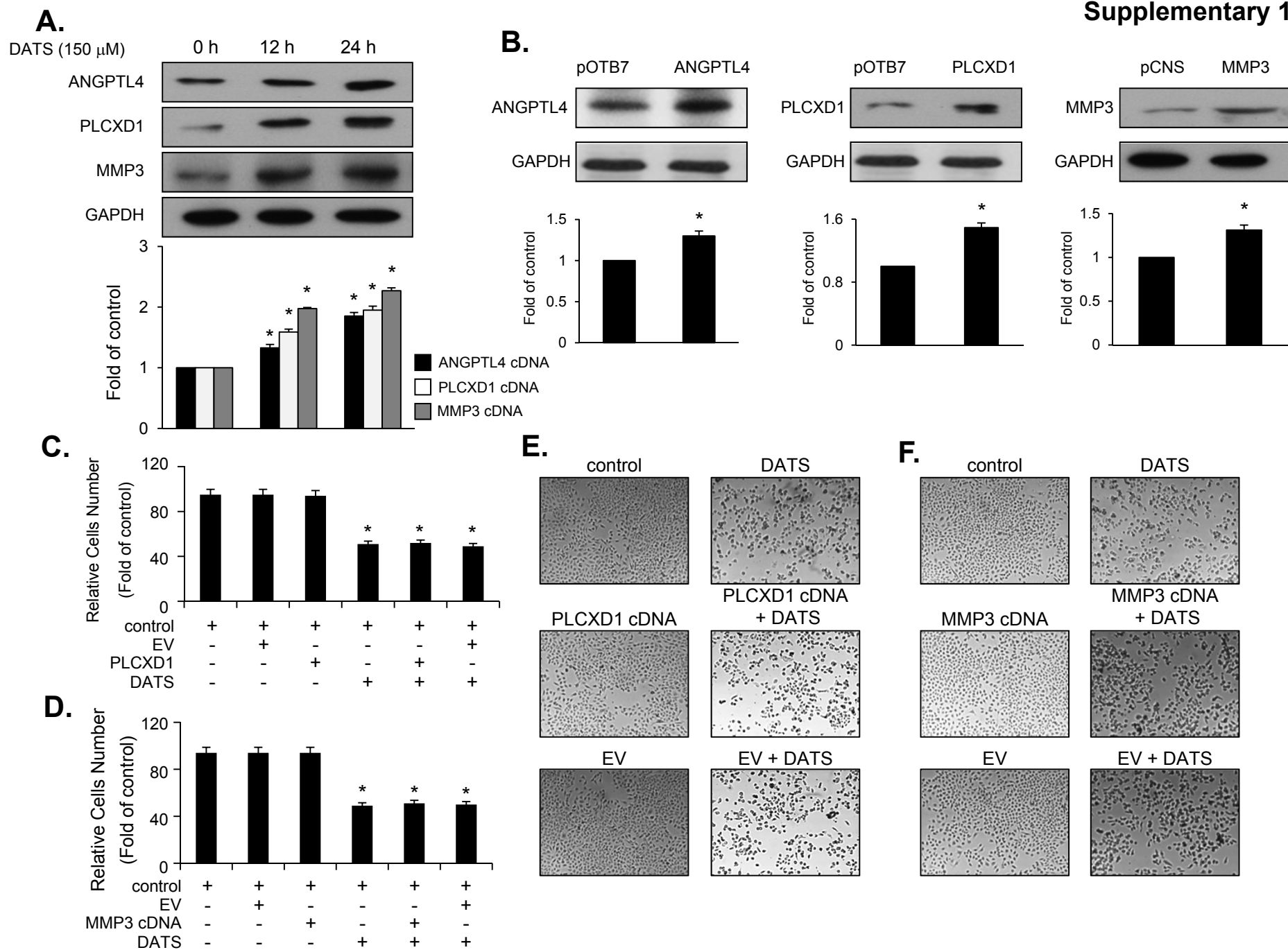

## Supplementary 2.

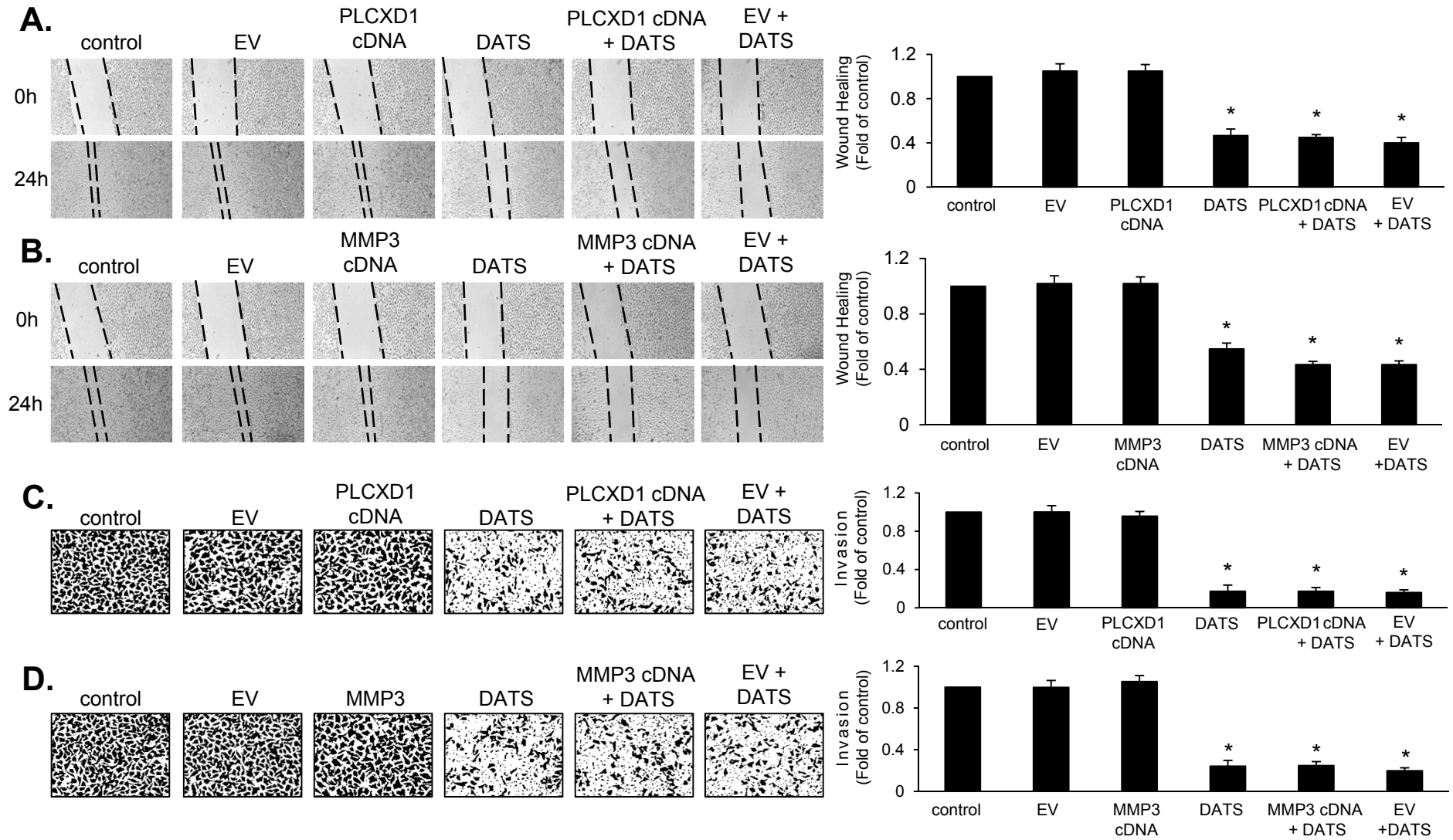

Supplement: ZFNR_A_1338918_Supplemental_data.zip [file zfnr_a_1338918_sm0516.zip › ZFNR_A_1338918_Supplemental data/DATS_EJ_Data_supplementary figures.pdf]
